# The Hidden Hitchhiker: Retrocaval Ureter Masquerading as Isolated Pelviureteric Junction Obstruction

**DOI:** 10.7759/cureus.115712

**Published:** 2026-09-03

**Authors:** Ajay Ramesh, Venkateshen Palanisamy, Roshan Yedulla Reddy, Hariharasudhan Sekar, Sriram Krishnamoorthy

**Affiliations:** 1 Urology and Renal Transplantation, Sri Ramachandra Institute of Higher Education and Research, Chennai, IND; 2 Urology, Sri Ramachandra Institute of Higher Education and Research, Chennai, IND

**Keywords:** congenital anomaly, pelviureteric junction obstruction, pyeloureteral reconstruction, retrocaval ureter, robotic pyeloplasty

## Abstract

The coexistence of true pelviureteric junction obstruction (PUJO) and a retrocaval ureter in a single, non-duplicated collecting system is exceptionally rare, and this case represents a "hidden retrocaval ureter," in which an upstream PUJO masked the classical radiological features, transforming the diagnosis into an unexpected intraoperative discovery. Our case highlights such a peculiar case, where a 70-year-old man with longstanding intermittent right loin pain was found to have right hydronephrosis and a serum creatinine of 1.7 mg/dL. Computed tomography showed a markedly dilated extra-renal pelvis resembling PUJO. The radiological report suggested a ureteric stricture at that level. Magnetic resonance urography also showed an abrupt medial narrowing at the L3-L4 level, but the ureter could not be seen passing behind the inferior vena cava (IVC). The abrupt medial kink raised suspicion of a retrocaval ureter. During robotic surgery, the ureter was found coursing behind the IVC, confirming a retrocaval ureter. Surprisingly, dividing this segment did not decompress the tense renal pelvis. Further dissection revealed a separate narrowing at the true PUJ, and urine was released only after the pelvis was opened above this obstruction. The PUJO had prevented urine from entering the retrocaval segment, which explained why it was not visible on preoperative imaging. Both narrowed segments were excised, the ureter was brought anterior to the IVC, and pyeloureteral reconstruction was performed over a double-J stent. This case is unusual not only because the two conditions occurred together but also because one obstruction concealed the other.

## Introduction

Pelviureteric junction obstruction (PUJO) is an anatomical or functional impediment to drainage from the renal pelvis into the proximal ureter. It is a commonly encountered cause of hydronephrosis and may become apparent in infancy, during evaluation of antenatal hydronephrosis, or much later in adulthood. The obstructive segment may be intrinsically narrow or aperistaltic, associated with a high ureteric insertion or periureteric fibrosis, or compressed extrinsically by a crossing vessel. In adult PUJO, crossing vessels followed by intrinsic stenosis, adhesions, and high ureteric insertion were the most common causes [1]. Clinical presentation varies from incidental hydronephrosis to intermittent flank pain, urinary infection, stone formation, and progressive deterioration of renal function.

Retrocaval ureter, also termed circumcaval ureter, is a different congenital entity. The ureter itself develops normally; the anomaly results from persistence of an abnormal embryonic venous segment, causing the right ureter to pass posterior to the inferior vena cava (IVC), usually near the third lumbar vertebra. Compression between the IVC and the vertebral column, together with angulation and periureteric fibrosis, may result in proximal hydroureteronephrosis. Type I disease typically forms a low loop with the classical S-shaped or fish-hook configuration and is more often obstructive. In contrast, the higher-loop Type II variant may show a gentler sickle-shaped course and less marked dilatation. This is an uncommon but surgically important venous-development anomaly [2].

Cross-sectional urographic imaging usually demonstrates the ureter passing behind the IVC, and computed tomography (CT) is commonly regarded as the most direct method of diagnosis. Magnetic resonance (MR) urography is useful when iodinated contrast is undesirable or renal function is impaired [3]. Nevertheless, the diagnosis can be missed when the retrocaval segment does not fill, when the anomaly is high, or when the proximal collecting system resembles a conventional PUJO. A fish-hook appearance may represent retrocaval ureter, but similar configurations can occur in PUJO or a duplicated system, and missed retrocaval ureter has been recognized after an unsuccessful pyeloplasty [4,5].

From an Indian perspective, the simultaneous presence of a genuine PUJO and a retrocaval ureter in a single, non-duplicated collecting system is rare. A 2025 report described this combination [6], and an older report documented it in association with a horseshoe kidney [7]. The present case is noteworthy because the upstream PUJO concealed the retrocaval segment on preoperative imaging, and both abnormalities were established only during robotic exploration.

## Case presentation

A 70-year-old man presented with right loin pain to our tertiary care referral teaching institution in South India. The discomfort had never been severe enough to prompt definitive evaluation. During this prolonged period, he had managed the episodes with analgesics and occasional empirical antibiotics. The patient had a history of right inguinal hernioplasty in 2025 and coronary artery bypass grafting in 2013.

An ultrasonogram obtained during the current evaluation demonstrated right hydronephrosis. A non-contrast CT of the kidneys, ureters, and bladder (KUB) showed a markedly dilated right renal pelvis and an appearance strongly suggestive of PUJO. A technetium-99m diuretic scan showed bilateral shrunken kidneys with significant tracer retention in the right kidney, with split renal function of 55% on the left and 45% on the right. Overall glomerular filtration rate (GFR) was 40.3, left kidney GFR was 22.1 mL/min, and right kidney GFR was 18.2 mL/min. Serum creatinine was 1.7 mg/dL. Because renal function was mildly impaired, we obtained MR imaging with angiographic/urographic assessment rather than a conventional contrast CT urogram. This again demonstrated a hugely dilated extrarenal pelvis with an abrupt acute kink at approximately the L3-L4 level. The radiological report suggested a ureteric stricture at that level (Figure 1c). Although the abrupt medial kink raised suspicion of a retrocaval ureter, no urine-filled ureteric segment could be demonstrated posterior to the IVC on the available sequences. The working interpretation was therefore a right PUJO associated with a large, redundant, and baggy extrarenal pelvis (Figures 1a, 1b). A right robot-assisted pyeloplasty was planned.

**Figure 1:**
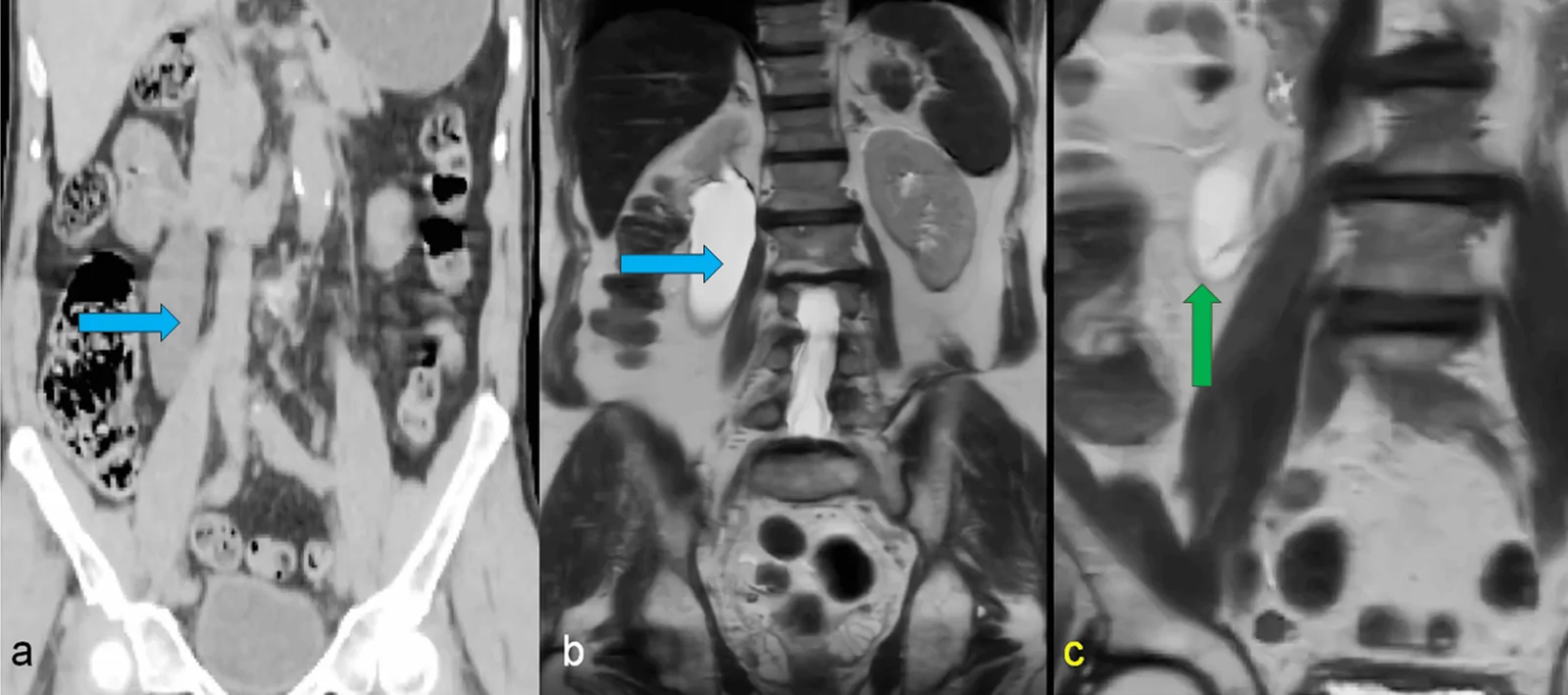
Preoperative imaging evaluation. (a) Coronal non-contrast CT showing marked right hydronephrosis (APRPD: 2.4 cm) with a dilated extrarenal pelvis and proximal ureteric segment with an abrupt medial kink and tapering at the L5 vertebral level (blue arrow). (b) Coronal MR urography demonstrating a markedly dilated renal pelvis with an abrupt medial kink at the L3-L4 level. (c) Magnified MR image showing the acute medial kink near L3-L4 (green arrow); the retrocaval segment was not visualized. APRPD: anterior-posterior renal pelvic diameter; CT: computed tomography; MR: magnetic resonance

Intraoperative diagnostic dilemma

At robotic exploration, extensive bowel adhesions to the peritoneal wall were encountered and carefully released. Identification of the renal pelvis was difficult. Progressive mobilization demonstrated the markedly dilated renal pelvis and proximal ureter.

The PUJ was then defined. The ureter proximal and distal to the apparent PUJ region was dissected, and the proximal segment was looped with a vessel loop (Figure 2b). As the dissection proceeded medially, the ureter could not be followed; hence, the IVC was delineated and mobilized. A peristaltic tubular structure was identified emerging from behind the IVC and was confirmed as the distal ureter. This distal segment was separately looped (Figure 2c). The operative field now demonstrated the proximal and distal ureteric segments with an intervening portion coursing posterior to the IVC (Figure 2d), establishing the first intraoperative surprise: a retrocaval ureter that had not been demonstrated on either CT or MR imaging.

**Figure 2:**
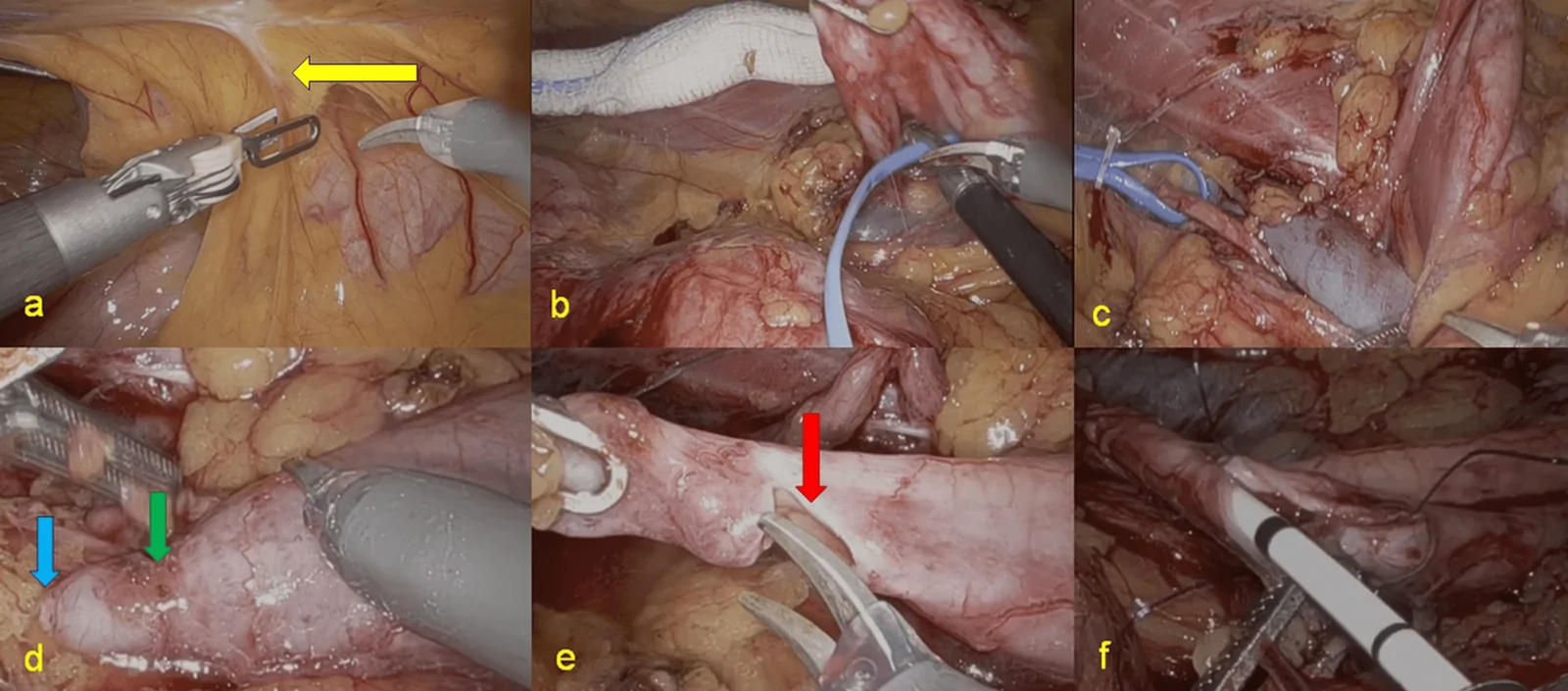
Robotic findings and reconstruction. (a) Dense adhesions over the operative field (yellow arrow). (b) Proximal ureter isolated with a vessel loop. (c) Proximal and distal ureteric segments defined in relation to the IVC. (d) Retrocaval course confirmed, with the two ureteric segments indicated by blue and green arrows. (e) Separate narrowing at the true PUJ (red arrow). (f) Posterior pyeloureteral reconstruction over a double-J stent. IVC: inferior vena cava; PUJ: pelviureteric junction

The second surprise

The operative anatomy initially appeared explainable by the retrocaval segment alone. The ureter was divided to release it from behind the IVC. In an isolated obstructive retrocaval ureter with a freely communicating dilated pelvis, division of the ureter would be expected to release urine and decompress the proximal collecting system. In this patient, however, there was no gush of urine and the markedly ballooned renal pelvis remained tense.

Careful inspection of the renal pelvis revealed a separate constricted segment at the true PUJ. The renal pelvis was opened proximal to this narrowing. Urine then gushed out, and the previously tense pelvis collapsed immediately (Figure 2e). This provided direct intraoperative confirmation of a second and independent obstructive lesion: a genuine PUJO located proximal to the retrocaval ureter. Thus, the patient had two congenital obstructive abnormalities on the same side in a single collecting system. The findings suggest that the PUJO had prevented urine from entering and distending the retrocaval ureteric segment, explaining why T2-weighted MR sequences did not display the expected urine-filled course behind the IVC (Figure 1c) and why the case had masqueraded radiologically as isolated PUJO.

Robotic reconstruction

The retrocaval portion of the ureter was completely freed, and the abnormal or narrowed segment was excised. The distal ureter was transposed anterior to the IVC and spatulated widely. The obstructed PUJ and redundant pelvic segment were excised, and a direct pyeloureteral reconstruction was fashioned between the renal pelvis and healthy ureter. The posterior wall was sutured first with particular attention to mucosa-to-mucosa approximation, avoidance of intervening knots or suture material, and preservation of a wide dependent lumen. A double-J (DJ) stent was passed across the reconstruction (Figure 2f), after which the anterior wall was completed.

The procedure was otherwise uneventful, the drain was removed and the patient was discharged on postoperative day (POD) 2, and the urinary catheter was removed on POD 5. Port site staplers were removed on POD 7. Histopathological examination (HPE) of the excised PUJ showed pelviureteric tissue with fibrosis and mild chronic inflammation.

The patient is now on routine follow-up. He was reviewed on the sixth postoperative week and posted for cystoscopy + right DJ stent removal + retrograde pyelogram. Right DJ stent removal was performed, retrograde pyelogram showed free flow of contrast passing to the renal pelvis, and postoperatively, serum creatinine reached a baseline of 1.2.

## Discussion

This case demonstrates why a presumed PUJO should not end the anatomical inquiry when the ureter shows an abrupt medial deviation or an unusual kink near the IVC. The diagnostic error was not simply a failure to recognize a classical fish-hook sign. The retrocaval segment was functionally excluded from the urine column by the upstream PUJO. As a result, neither the non-contrast CT nor the MR study could outline the ureter behind the IVC. The enormous extrarenal pelvis and true PUJ narrowing dominated the images, while the distal anomaly remained silent.

A standard pyeloplasty performed without tracing the ureter to its distal course could have left the retrocaval segment untreated, an issue described in missed cases presenting after failed surgery [5]. The age and duration of symptoms also merit attention. Although retrocaval ureter classically becomes symptomatic in younger adults, obstruction is variable and may remain clinically tolerated for many years. This patient reached 70 years after almost two decades of intermittent symptoms, suggesting a slowly progressive or intermittently significant obstruction. His mildly elevated creatinine, along with the radiographic findings, further strengthened the need for definitive anatomical correction while avoiding assumptions based solely on a common radiological diagnosis.

The principal learning point is not that every PUJO requires extensive caval dissection. Rather, a high index of suspicion is warranted when imaging shows a disproportionately large extrarenal pelvis, with failure to visualize the ureter below the apparent PUJ, or discordance between the presumed level of obstruction and the observed anatomy. Delayed urographic imaging, three-dimensional reconstruction, MR urography, and retrograde pyelography may clarify uncertain cases [4,5]. When imaging remains equivocal, minimally invasive exploration should include identifying the ureter relative to the IVC before committing to reconstruction. In a similar case of retrocaval ureter, robot-assisted ureteroplasty and direct pyeloureteral anastomosis showed good short-term anatomical outcomes [8-11].

## Conclusions

Retrocaval ureter and PUJO can coexist in the same kidney and the same single collecting system. In the present case, true PUJO prevented urine from entering the retrocaval ureter, obscuring the classic radiological appearance and leading to a convincing preoperative diagnosis of isolated PUJO. The double obstructive pathology became evident only through systematic robotic dissection and intraoperative testing of the level of obstruction. Surgeons should remain alert to a retrocaval course when a right-sided PUJO has an abrupt medial kink or a poorly visualized distal ureter.
